# Compartment models for the electrical stimulation of retinal bipolar cells

**DOI:** 10.1371/journal.pone.0209123

**Published:** 2018-12-17

**Authors:** Frank Rattay, Hassan Bassereh, Isabel Stiennon

**Affiliations:** Institute for Analysis and Scientific Computing, Vienna University of Technology, Wiedner Hauptstrasse, Vienna, Austria; Doheny Eye Institute/UCLA, UNITED STATES

## Abstract

Bipolar cells of the retina are among the smallest neurons of the nervous system. For this reason, compared to other neurons, their delay in signaling is minimal. Additionally, the small bipolar cell surface combined with the low membrane conductance causes very little attenuation in the signal from synaptic input to the terminal. The existence of spiking bipolar cells was proven over the last two decades, but until now no complete model including all important ion channel types was published. The present study amends this and analyzes the impact of the number of model compartments on simulation accuracy. Characteristic features like membrane voltages and spike generation were tested and compared for one-, two-, four- and 117-compartment models of a macaque bipolar cell. Although results were independent of the compartment number for low membrane conductances (passive membranes), nonlinear regimes such as spiking required at least a separate axon compartment. At least a four compartment model containing the functionally different segments dendrite, soma, axon and terminal was needed for understanding signaling in spiking bipolar cells. Whereas for intracellular current application models with small numbers of compartments showed quantitatively correct results in many cases, the cell response to extracellular stimulation is sensitive to spatial variation of the electric field and accurate modeling therefore demands for a large number of short compartments even for passive membranes.

## Introduction

Bipolar cells (BCs) are second order neurons of the retina that receive synaptic input from photoreceptors and transmit signals to ganglion cells. They are among the smallest neurons in the nervous system. Upwards of 10 types of BCs each were reported for various vertebrates [1–3]. There is significant variation in cell size and morphology both between species and between BC types in the same animal.

Types of BC ion channels were first detected and quantified in fish, as their comparatively large, spherical axon terminal provides better access for measurement than those of other species. This led to the development of an early single compartment model [4]. The axons of other vertebrate BCs feature a large number of small terminals which connect to ganglion cells in various depths of the inner plexiform layer. The location of these synapses influences the axon length and shape and thus the BC type. The resulting differences in neurite shapes and lengths may impact the suitability of a given computer modeling approach to the BC type in question.

BCs contain sodium, potassium, hyperpolarization-activated cyclic nucleotide-gated (HCN), and calcium ion channels. Ion channel types and densities vary depending on the cell and region within the cell [5–11].

For many years only amacrine and ganglion cells were considered to be able to generate action potentials (spikes) in the retina, but more recently spiking has also been observed in BCs [12, 13] and is likely mainly due to sodium channels in the axon [14–16]. Two bipolar cell types of the magnocellular pathway in the primate retina, the diffuse bipolar cells DB3 and DB4, are involved in motion and flicker detection where high temporal resolution is needed. Their ability to generate sodium spikes supports the quick signal transport from photoreceptors to ganglion cells as, compared to graded potentials, a spike causes stronger synaptic activity at the BC terminal [17]. Even before BC spikes were discovered, several types of ion channels were recorded that make the BC membrane an active element [18–20]. These channels are likely also present in non-spiking cells.

Computer simulations of individual BCs have been used to model a variety of scenarios, from physiological synaptic input by photoreceptors [21, 22], to artificial stimulation by patch-clamp [4, 23] and extracellular electrodes [24–27]. The latter has applications in the design of retinal implants [28] where one of the great challenges is simultaneous stimulation of neighbored ON and OFF cells which hinders better spatial resolution. BCs of the ON type are active when light becomes brighter in contrast to OFF cells which release more neurotransmitter when local light intensity decreases. Simultaneous extracellular stimulation would confusingly signal that this region is both brighter and darker than the surrounding regions.

A common approach for simulating neurons is compartment modeling, which views a cell as comprising one or more functional units called compartments. These elements are often cylinders or truncated cones, whose interactions with the environment (i.e. the extracellular space and bordering compartments) are governed by equivalent electrical circuits representing the electrophysiological properties of the system.

Two examples for such circuit elements are the membrane capacitance, which usually has the same value per unit area in all compartments, as well as variable conductivities representing ion channels. Compartments connect to their neighbors by a longitudinal resistance determined mainly by their geometric properties such as length and diameter [29, 30]. The following subsections provide an overview over the most commonly used BC compartment models.

### Single compartment model (SCM)

In the simplest case, the entire cell is modeled using just one compartment. A major advantage of this and to a lesser degree all models with a low compartment number is the reduced need for computational resources and time. However, there are a number of drawbacks that make it badly suited for a range of tasks. An example is the forced homogeneity in cellular properties such as ion channel density. Additionally, since the cell’s location is represented by a single circuit node at the center of its compartment, the model of the neuron cannot reflect any of the effects of the spatial arrangement of its different parts.

These limitations notwithstanding, SCMs have been successful in modeling a variety of phenomena. Ishihara et al. (1998) used a system consisting of rod and cone photoreceptors connected to BCs where each cell was represented by a SCM [21]. This simulation was able to reproduce the effect of hyperpolarizing currents on the response of rod-dominated ON BCs under different light intensities. Publio and coworkers [31] studied the role of gap junctions in enhancing the dynamic range of the retina by modeling some of its cells (including BCs) as SCMs. A recent study [32] investigated the roles of AMPA receptor kinetics in OFF-BCs in temporal coding and synaptic transmission from cone cells.

### Two compartment model (TCM)

A step up from the SCM in complexity, the TCM cell’s separate compartments allow each to have different properties such as location and ion channel distribution. Ishihara and coworkers [22] analyzed the impact of ionic currents on light responses using a model consisting of a soma and an axon terminal compartment connected by a resistor representing the axon. This is sufficient for scenarios in which passive current flows through the axon have a much stronger effect on the BCs overall behavior than axonal transmembrane currents which are not accounted for in this model.

The TCM has proved useful for modeling BCs with a passive membrane [23, 33] as well as in simulations of extracellular stimulation when the transmembrane voltage is recorded at the terminal [26].

### Multi compartment model (MCM)

MCMs consist of at least three compartments. They are often 3D models comprising many cylindrical or cone shaped segments designed to closely match the morphology of a real cell. The template is obtained by recording an explanted BC with one of several tracing techniques such as tagging the neuron with fluorescent markers before imaging it with a two photon [34], confocal [35] or electron microscope [36].

Using a MCM, Oltedal and coworkers studied the effect of axon morphology on the passive signal attenuation between soma and axon terminal in rat BCs and evaluated variations of electrical parameters such as intracellular and transmembrane resistivity for several cells [37]. Short segments were needed to take the diameter variation along the cell into account. Especially for passive membrane models, several segments within dendrites and axon can be combined into equivalent cylinders in order to reduce the number of compartments without losing numerical accuracy. Each equivalent cylinder has the surface and the intracellular resistance of the fiber segment it represents [38]. The MCM type was also used to investigate the response of ON and OFF BCs to extracellular stimulation [38], to analyze the impact of calcium current reversal on neurotransmitter release during subretinal stimulation [39] and to simulate sodium spikes in a BC [40]. Passive filtering by the cell membrane in the terminal during extracellular stimulation was investigated using both a TCM and a morphologically realistic MCM resulting in cutoff frequencies of 895Hz and 717Hz, respectively [26].

### Conversion of MCMs from 3D to 2D

**Fig 1:**
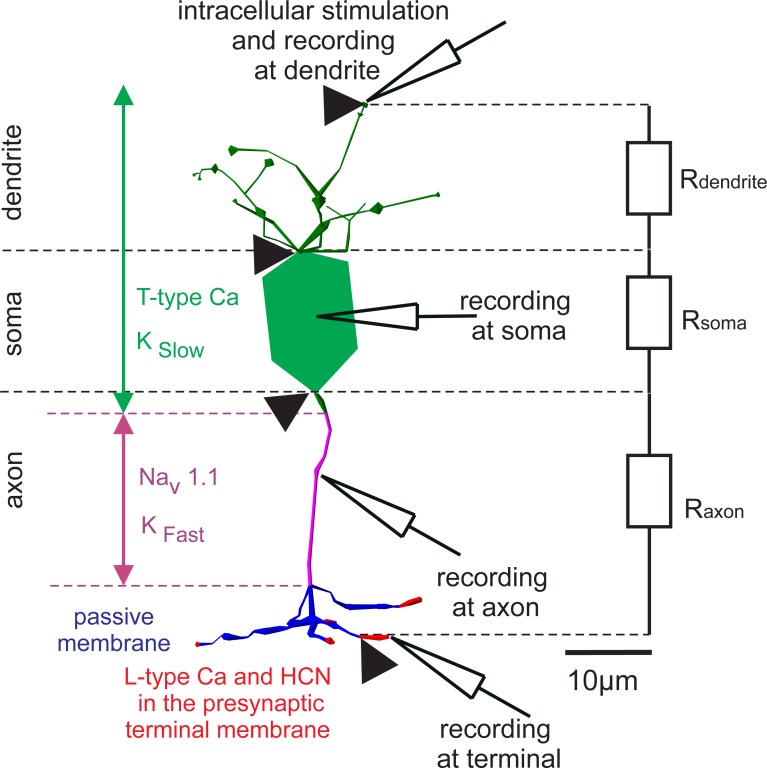
Functional segments with the main ion channel types of a DB4 BC consisting of 117 compartments. Dendrites, soma, and axon hillock contain Cav3.1 and slow potassium channels highlighted in green. Nav1.1 sodium channels and fast-type potassium channels are distributed on the axon (purple); the blue parts represent the passive parts of the axon. The terminals (red) contain HCN and L-type Cav1.4 channels. The diagram shows a typical simulation task where the BC was stimulated via synaptic input current and the response was recorded at 4 sites. Cell geometry and experimental data were taken from [9].

Although three-dimensional neuron models based on recorded cells are available in internet databases, these sources still contain rather few retinal BCs. To address this issue a Matlab method was developed to generate 3D cell geometries [41] based on some of many available BC 2D morphologies [36, 42]. Encke’s method [41] determines the positions and local diameters of the neuron parts from the 2D image and estimates the missing third dimension with normally distributed random values. Conversely, a 3D model can be simplified to a 2D model if desired in a simulation, e.g. by projection to a suitably chosen plane. This is the case for the cell shown in Fig 1, where the total length of all dendrites in 3D was 126.2 μm and 98.98 μm in 2D.

As indicated in the previous paragraphs, the required number of compartments and properties of a model depend on the specific question it is designed to address. If different models can be shown to produce similar results, it is usually desirable to choose the least resource intensive one, i.e. generally the one with fewer compartments. Another application for lower complexity models are situations in which detailed cell tracings and thus 3D MCM are not available, as is often the case with BCs.

Three common stimulation types were investigated in this study; (i) synaptic input at the dendrite by photoreceptors, which was simulated with intracellular current injection at the dendrite; (ii) soma stimulation via microelectrode, a commonly used method in experiments and (iii) extracellular stimulation via microelectrode, which is a simple model for BC stimulation with retinal implants. The aim was to demonstrate that it is possible to achieve satisfactory results with a small number of compartments for a range of stimulation tasks, independent of the type of BC.

## Material and methods

The four models investigated in this study were one-, two-, four- and multi compartment cells. Their properties were based on a macaque retinal DB4 BC, for which ion channel distribution and 3D morphology were recorded and reconstructed [9]. All ion channels were simulated with Hodgkin-Huxley kinetics [43].

For all models, the membrane capacitance C_m_ was 1 μF/cm^2^ [9], the intracellular resistivity 0.1 kOhm.cm and the extracellular resistivity 1 kOhm.cm. The leak current conductance gL was set to 0.033 mS/cm^2^ [9], the lowest of several reported values [23, 26, 37, 38]. Passive BCs were simulated by removing all ion channels except for the leak conductance. All simulations used Python 2.7 and were run at 31°C [30].

### Multi compartment model (MCM)

The 3D morphology of a DB4 cell was approximated using 117 compartments comparable to an earlier simulation [40]. The spherical soma was replaced with a cone-cylinder-cone combination that better fit the shape of the original cell. Five types of ion channels were located on different parts of the cell (Fig 1).

T-type Cav3.1 channels were homogeneously distributed (specific conductance gCa = 1 mS/cm^2^) in the soma, dendrite, and axon hillock compartments; for the kinetics see [44].

The potassium channels can be divided into a fast and a slow type. The fast one was located in the sodium band (the part of the axon containing the sodium channels) with gKfast = 2 mS/cm^2^, while the slow one was located at soma, dendrite, and axon hillock with gKslow = 2.4 mS/cm^2^. Potassium conductance values were fitted to voltage clamp experiments in [9]. The kinetics of potassium channels were based on the original Hodgkin-Huxley potassium channel model including a voltage offset and a tau correction factor, τ_corr_ to fit kinetics seen in experiments. The fast potassium channel type located in the axon was shifted by a voltage offset of 5mV (causing channels to open at a 5mV higher membrane voltage) and τ_corr_ = 5 (5 times slower than standard Hodgkin-Huxley potassium channel), while the slow channel had no voltage shift and τ_corr_ = 8.

The sodium channel (Nav1.1) density had two peaks on the axon represented by gNa = 650 and 1000 mS/cm^2^ that were at a distance of 12.3 μm and 22.7 μm from the soma, respectively [9]. In the MCM, axon stimulation was applied to the most excitable compartment with gNa = 1000 mS/cm^2^. Sodium channel kinetics were based on [45, 46]. To determine the inactivation variable h, data from a 5 Hz sinusoidal current injection experiment was fitted by τ_corr_ = 4 [9].

The kinetics and conductances of L-type calcium and HCN1 channels at the terminals (marked in red in Fig 1) fit data of [9, 47]. Their respective conductances were 11.9 and 3.52 mS/cm^2^. The kinetics of calcium L-type mechanisms were based on [39] and of those of the HCN channels on [48].

Further details on channel kinetics are described in the appendix.

### Single compartment model (SCM)

The SCM had a surface area equal to the total surface of the real cell i.e. 650 μm^2^. All ion channels present in the MCM were included with the same kinetics but with averaged conductivities of gNa = 23.52, gKfast = 0.1, gKslow = 1.83, gCa3.1 = 0.76, gCa1.4 = 0.38 and gHCN1 = 0.11 mS/cm^2^.

### Two compartment model (TCM)

**Fig 2:**
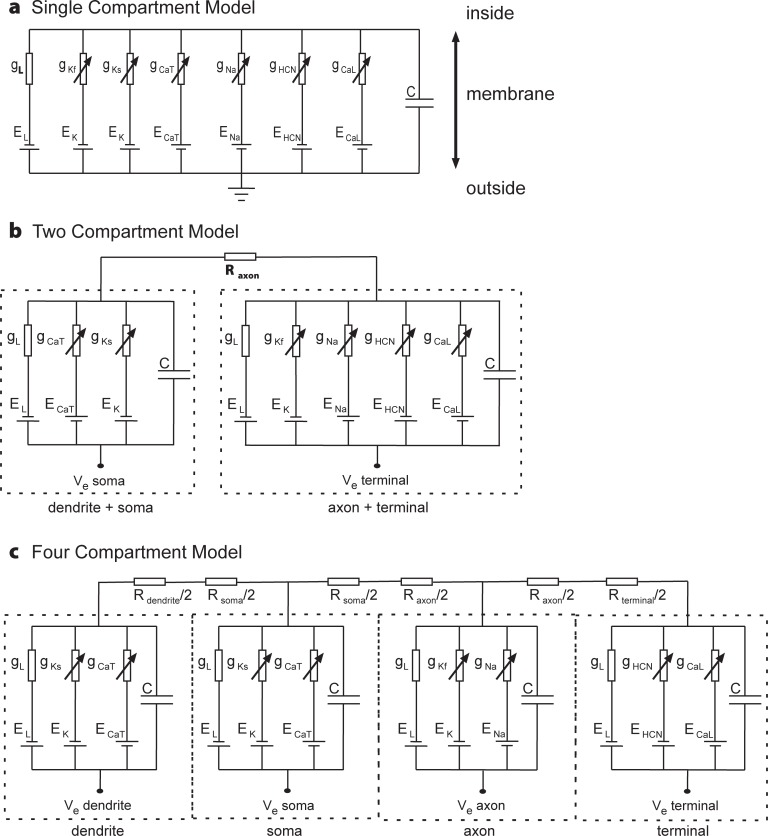
**Equivalent electrical circuits for a) SCM, b) TCM, c) FCM**. The presented simulations were based on modified data from a type 4 BC (See Methods).

The TCM consisted of a somatodendric compartment and an axonal terminal, connected via a resistance representing the axon (Fig 2). To be comparable with other models, the TCM surface was again set to 650 μm^2^. We cut the geometry of Fig 1 at the center of the axon and added half of the axon surface (total 43 μm^2^) to the soma resulting in a soma surface of 512 μm^2^ and a terminal surface of 138 μm^2^. Axon length and diameter were 27.3 and 0.5 μm. The Kslow and Cav3.1 channels were assumed to be located in the somatodendric compartment with averaged conductances of 2.32 and 0.96 mS/cm^2^, respectively. The averaged conductances of Nav1.1, Kfast, Cav1.4 and HCN1 channels located at the terminal were 110.95, 0.475, 1.81 and 0.535 mS/cm^2^. As in the SCM, the kinetics of the ion channels were not changed from the MCM.

### Four compartment model (FCM)

The FCM divided the cell into four cylindrical compartments representing dendrites, soma, axon, and terminal. The surface area of each compartment was equal to the total surface area of all compartments making up that cell region in the MCM. The diameters of the FCM compartments were the average of the diameters of all corresponding MCM compartments. The areas of dendrite, soma, axon, and terminal were 135.45, 355.13, 138.39, and 20.8 μm^2^, and the diameters 0.6, 10.7, 0.62, and 0.6 μm, respectively. Given the cylindrical shapes, the compartment lengths were calculated from the surfaces as 72.39, 10.56, 71.04 and 11.03 μm. For extracellular stimulation those lengths were used to calculate the intracellular resistances. The corresponding locations at which the four Ve values were calculated were determined by the centers of the structures they represented.

The axon contained Nav1.1 and fast potassium channels with conductances of 110.5, and 0.47 mS/cm^2^, respectively. Cav3.1 and slow potassium channels were located on the soma and dendrite with conductances of 1.0 and 2.4 mS/cm^2^. Cav1.4 and HCN1 channels were present on the terminal with conductances of TCM.

### Intracellular vs. extracellular stimulation

Kirchhoff's law applied for the n-th compartment, e.g. for FCM according to Fig 2C, includes a capacitance current, ionic transmembrane current, intracellular currents to the left and right neighbor compartments resulting in

$$C_{n}\frac{{d(V}_{i,n}-V_{e,n})}{dt}+I_{ion,n}+\frac{V_{i,n}-V_{i,n-1}}{R_{n}/2+R_{n-1}/2}+\frac{V_{i,n}-V_{i,n+1}}{R_{n}/2+R_{n+1}/2}=I_{stim,n}$$ (1)

with intracellular potential *V*_*i*_, extracellular potential V_e_, intracellular resistance *R*, membrane capacity *C* and possible injected current *I*_*stim*_. As the first and last compartment has only one neighbor, Eq 1 is reduced by one term. The transmembrane voltage is defined as

$$V_{m}=V_{i}-V_{e}$$ (2)

For intracellular stimulation *V*_*e*_ = 0 and consequently *V*_*m*_ = *V*_*i*_. When starting at rest the ion currents as well as currents to the neighbor compartments are zero and (1) is reduced at stimulus onset to

$$C_{n}\frac{{dV}_{m,,n}}{dt}=I_{stim,n}$$ (3)

As an example, depolarization by intracellular soma stimulation requires a positive pulse and this first activity depolarizes the neighboring compartments via intracellular current flow, thus depolarizing the whole cell. Note that usually only one of the compartments is stimulated via current injection.

The excitation mechanism for extracellular stimulation is more complicated as the applied field has a direct stimulating effect along the whole cell [29] which can be seen as virtual individual current injection at each of the compartments [40, 49]. In order to find these currents (2) is rearranged to *V*_*i*_ = *V*_*m*_ + *V*_*e*_ and (1) reads as

$$C_{n}\frac{{dV}_{m,n}}{dt}=-I_{ion,n}+\frac{V_{m,n-1}-V_{m,n}}{R_{n}/2+R_{n-1}/2}+\frac{V_{m,n+1}-V_{m,n}}{R_{n}/2+R_{n+1}/2}+(\frac{V_{e,n-1}-V_{e,n}}{R_{n}/2+R_{n-1}/2}+\frac{V_{e,n+1}-V_{e,n}}{R_{n}/2+R_{n+1}/2})$$ (4)

All terms in (4) are currents and the term in brackets is the virtual injected current that represents the stimulating influence of the electric field on compartment *n*. In contrast to intracellular stimulation, the sum of the virtual currents is zero. This implies that, contrary to intracellular stimulation, an extracellularly placed microelectrode causes polarized and depolarized regions simultaneously. Note that the key stimulating elements of the electric field that dominate the virtual currents in (4) are not the actual *V*_*e*_ values but *V*_*e*_ differences between compartments.

## Results

### Passive vs. active cell membrane

Only a small fraction of BC types in mammals generate spikes, e.g. 2 in macaque [9]and 3 in mouse retinas [10]. For all other types, a passive cell membrane with a low membrane conductance determines the stimulus/response relationship and is responsible for the model-typical large time constant of 30 ms.

**Fig 3:**
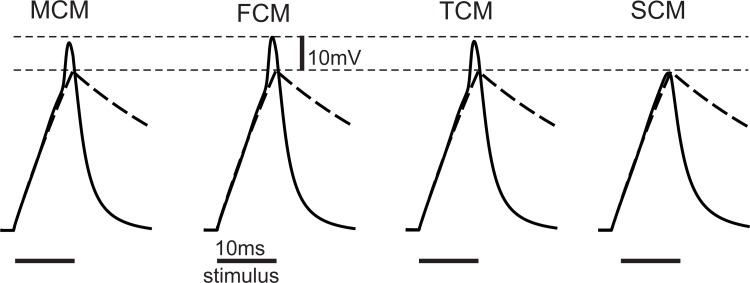
Transmembrane voltage over time in the soma for somatic stimulation. A 35pA/10ms pulse caused spikes in MCM, FCM and TCM, whereas no spike developed when the axonal channels were included in the soma compartment. All models showed a comparable performance for passive membranes (dashed lines).

A current pulse of 35 pA with a duration of 10 ms was applied at the soma to both passive and active versions of the compartment models. The resulting somatic transmembrane voltages for each case are displayed in Fig 3. Spiking was not activated when the sodium ion channels are shifted from the axon to the dendrite soma region, as is the case in SCM. In a variation test of TCM where the axonal ion channels were shifted from the terminal to the soma compartment, the TCM response was not distinguishable from that of the SCM.

The higher efficiency of axonal sodium channels is based on the resistance between soma and axon which allows a larger transmembrane voltage increase by the local sodium current influx during the development of the spike. A rough quantitative approach is possible when soma and axon are seen as single (separated) compartments with the same number of sodium channels. After stimulus end, the main Eq (1) can be reduced to

$$\frac{{d(V}_{i,n}-V_{e,n})}{dt}=-I_{ion,n}/C_{n}$$ (5)

where n represents either the soma or the axon and capacitance Cn is proportional to the surface of soma and axon, respectively. Using FCM data, the same (negative) sodium current resulted in a membrane voltage increase ratio in axon vs. soma of (soma surface)/(axon surface) = 355.13/138.39 = 2.56. This extreme ratio demonstrated that the easier axonal excitation was reduced by the finite resistance between soma and axon. Notably, the advantage of such a resistance is evident in most neurons where the axonal region with high sodium channel density is separated from the soma at least by a short segment, e. g. axon hillock (compare the first green axon segment in Fig 1 which is without sodium channels [9], [40]).

### MCM vs. FCM and axon length impact on the transmembrane voltage

**Fig 4:**
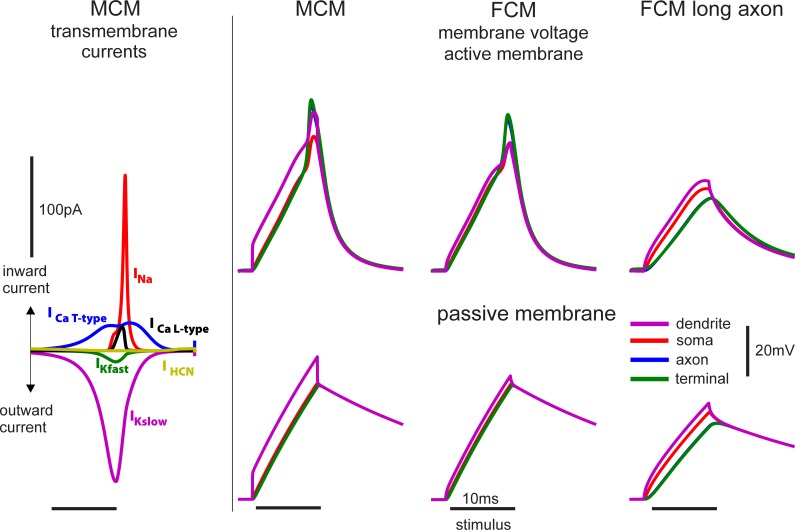
Simulated responses to intracellular stimulation at dendrite. Left) Transmembrane currents in the suprathreshold case for the active model. The T type calcium current as well as the slow potassium current (both distributed in dendrite and soma, see Fig 1) were activated before the sodium current. Inward calcium and outward potassium current canceled each other out and therefore didn’t affect the timing of the sodium current in either FCM or MCM. A large sodium current peak determined whether a response is above threshold. Right) The transmembrane voltage as recorded at the four standard locations (Fig 1). The 35 pA, 10 ms pulse caused a weaker response in the long axon model due to the increased surface.

The main difference in geometry between mammalian BC types is the axon length. To investigate the impact of different axon dimensions on model behavior, a new version of the FCM was created by tripling the axon length (including the branching part) from 97.09 to 213.27 μm. A passive and active version of this new model, the original FCM and MCM were subjected to intracellular stimulation of 35 pA for 10 ms at the dendrite tip. Fig 4 shows dendritic transmembrane currents for the MCM as well as the resulting membrane voltages in axon, dendrite, soma and terminal of the different models. The positions of the stimulating and recording electrodes in the MCM are depicted in Fig 1.

There were several differences in the membrane voltages between the models. Both active and passive MCM showed a notably higher transmembrane voltage during stimulation than the FCM. The dendrite tips in natural cells (and the MCM) are quite thin, which simultaneously increases the resistance between adjacent compartments and decreases their surface area. This means that the charge introduced by the electrode was distributed across less surface area within a compartment, resulting in a higher local transmembrane voltage compared to the FCM, where it was only slightly elevated. There was little difference between the transmembrane voltages of soma, axon and terminal in the individual models, with an exception of the long axon FCM. Axon and terminal voltage coincided due to small transmembrane currents in the passive part of the axon.

After a stimulation-induced offset in the active case, the MCM and the unmodified FCM showed comparable spikes, with the exception of the higher dendritic voltage in the MCM. The spike started out in the axon due to higher sodium channel activity and backpropagated to the soma and dendrites. The long axon FCM showed a generally lower membrane voltage in all compartments and no spikelet.

The voltages in the passive versions of the MCM and original FCM also matched quite well, while the voltages in the long axon model were again lower.

The longer axon increased axonal resistance and loss of signal through the larger neurite surface. To elicit the same response as in the original model, a higher stimulation intensity was needed in the larger cell. This means that a FCM developed for one kind of BC should not be used for a different type without adaptations.

### Maximum of membrane voltage for long pulses

**Fig 5:**
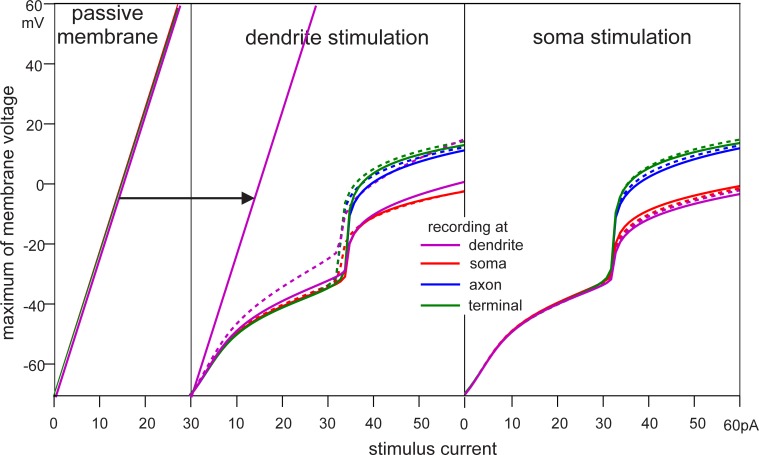
Passive vs. active response to 100 ms pulse. Left) Maximum transmembrane voltage over pulse amplitude for the passive MCM under dendritic stimulation. Recordings in soma, axon, and terminal compartments of the MCM, show the same linear behavior. Comparisons of FCM and MCM under dendritic (middle) and somatic (right) stimulation. Solid lines represent FCM and dashed lines the MCM. FCM responses were close to those of the MCM, with a maximum difference of less than 3% except for the case of current injection and recording in the dendrite.

Active and passive versions of both FCM and MCM were subjected to 100 ms current injections of varying intensity. The maximum membrane voltages were recorded in dendrite, soma, axon and terminal for somatic as well as dendritic stimulation and are displayed in Fig 5.

It was already demonstrated that all four passive models show similar transmembrane voltages for soma stimulation (Fig 3). As the passive system is linear, the maximum magnitude of the resulting transmembrane voltages was proportional to the stimulation current independently of the number of compartments or the stimulation site.

For both somatic and dendritic stimulation, the maximum voltages measured at the recording sites were fairly similar for the active MCM and FCM. The only exception was dendrite voltage in dendritic stimulation, which was noticeably higher in the MCM. This is consistent with the dendritic transmembrane voltages seen in Fig 4.

The maximum voltage in the active model was lower than in the passive model due to repolarizing transmembrane currents. Above the threshold at 32 pA, the resulting spike caused a sharp increase in maximum voltage, which was nevertheless always lower than in the passive model for long stimulation durations.

### Sinusoidal stimulation

**Fig 6:**
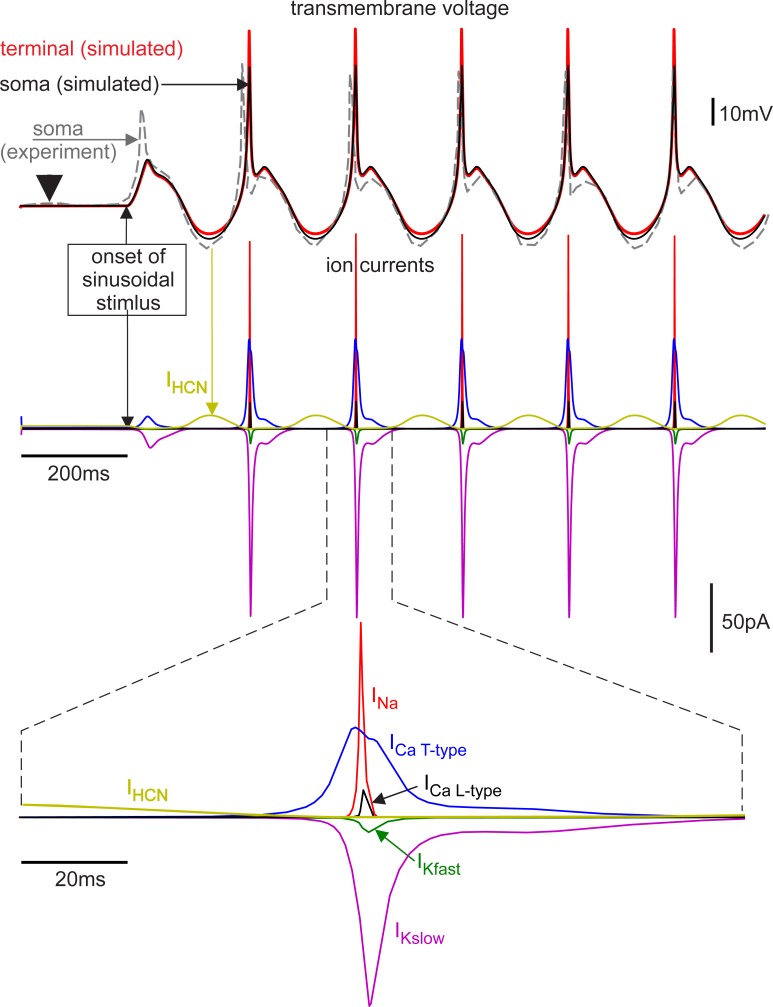
Simulated action potentials in response to 5 Hz 10 pA sinusoidal current injection at the soma correspond with experiments in timing and signal shape (top graph). The small first spike present in the experiment was missing in the model response as not every ion current type could be modeled with enough accuracy. Moreover, this spike may have been supported by current fluctuations across the membrane like the one before stimulus onset marked by arrowhead. The middle traces depict the ion currents including IHCN, which appears during hyperpolarization. Short sodium and calcium spikes in the terminal acted nearly simultaneously (bottom). Membrane voltage recorded at the soma is redrawn from Fig 9C of [9].

Experiments on BCs stimulated via sinusoidal current injection at the soma with a 10pA amplitude (Fig 9C in [9]) showed robust spiking (one spike per period) at 5Hz with a delay of 2.9±1.0 ms after the onset of each stimulus cycle. A similar response was seen in the multicompartment model (Fig 6), albeit with a somewhat longer delay.

The computer model demonstrated (i) an increase of membrane voltage in the terminal relative to the soma; (ii) a peak of the L-type calcium current in the presynaptic membrane appearing about 0.5 ms after the sodium current peak, which allowed quick synaptic activation; (iii) IHCN during the hyperpolarization phase of the sinus which supported spiking (marked by arrow in Fig 6, middle trace) and allowed for an increase of membrane voltage peak amplitude after the first sinus cycle in both experiment and simulation.

**Fig 7:**
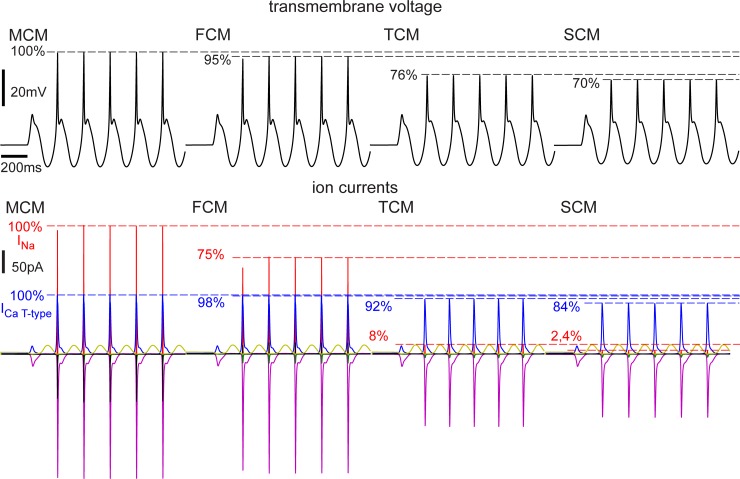
Action potentials and ion currents in response to 5 Hz sinusoidal current injection at the soma as in Fig 6 simulated with different compartment numbers. T-type calcium currents in dendrites and soma were not very sensitive to model reduction and lost up to 16% of amplitude (MCM vs. SCM). In contrast, the sodium current amplitude was dramatically reduced in TCM and SCM.

For comparison, the last MCM computer experiment (Fig 6) was repeated with FCM, TCM and SCM. A decrease of compartment numbers resulted in a decrease of spike amplitude (Fig 7). The main reason was the intracellular resistance between compartments which had no representation in the SCM and the allocation of sodium channels that were originally present in the axon to the soma compartment in the TCM. As it developed, the sodium spike needed a large early sodium current to pass through the axonal membrane, the resulting increase in membrane voltage leading to an activation of the gating process. To help with activation, axonal spike initiation zones in many neuron types were either thin themselves or located near a special thin segment, which caused greater resistance on both sides of the sodium band. This could not be modeled in the TCM and SCM and is the main reason why larger stimuli were needed in these models.

### Extracellular stimulation

#### Passive membrane

In extracellular stimulation, current is introduced via an electrode (a point source in this case that substitutes the current from the tip of a microelectrode) to the extracellular medium at some distance from the cell. Due to the constant resistivity of the extracellular medium, this results in a spherically symmetric potential gradient around the electrode. As a result, different points on a cell’s surface experience different extracellular potentials, depending on their distance to the electrode.

**Fig 8:**
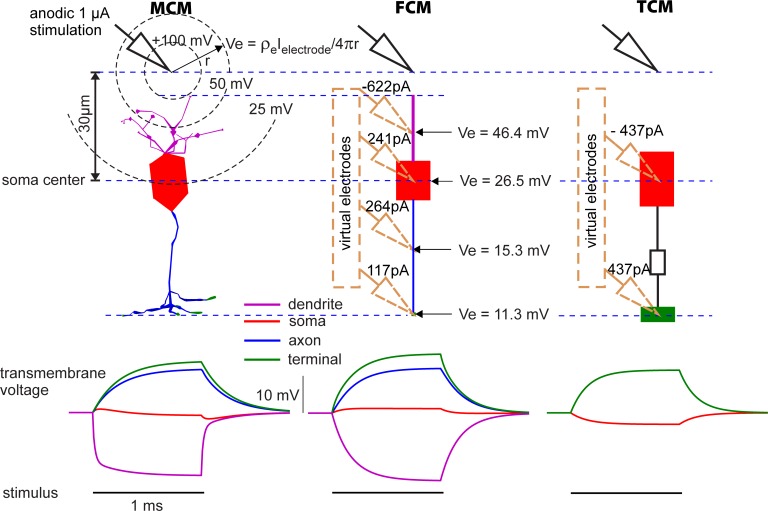
**Geometric layout (top) and passive membrane responses (bottom) of MCM, FCM, and TCM to anodic extracellular stimulation**. An anodic 1 μA pulse applied at the electrode tip 30 μm above the soma center generates spherical equipotential surfaces, changing the membrane voltage from the outside. For a distal ground, the extracellular potential Ve scales inversely with distance r from the tip of a microelectrode (see formula and dashed equipotential lines at MCM). Virtual electrodes are shown for FCM and TCM together with their stimulus amplitudes that are calculated according to Eq 4 (method section). Membrane voltage time courses for the selected BC regions depend on the number of compartments.

The sudden presence of a location dependent external electric field after the start of the stimulation causes capacitive currents through the cell membrane, which leads to a potential difference between different points in the interior of the cell. If the stimulation intensity stays constant, intracellular currents flow within the small BC until all internal points have assumed almost the same potential again. This takes more time for long, thin neurites that are oriented roughly perpendicular to the equipotential surfaces (Fig 8). The reason for this is the higher cytoplasmic resistance and the greater potential gradient over their lengths. If few or no charged particles pass the membrane, as is the case in passive models and shortly after stimulation onset even in active models, the average membrane voltage remains the same as before the stimulation. Since the entire interior of the cell is (almost) at the same potential after charge equilibration and the location dependent external field is still present during stimulation, the transmembrane voltage depends on location as well. ln anodic stimulation, the external potential close to the electrode is rather high and the potential far from the electrode rather low compared to the potential in the interior of the cell. For this reason, cell regions closer to the electrode are hyperpolarized and distant regions depolarized (see Fig 8); this is reversed for cathodic stimulation; for details see [40]. The transmembrane voltages of axon and terminal change slower than that of the short and thick soma (lower part of Fig 8). This simulation uses only passive models, if active channels were present they would influence the membrane voltage as well.

A crucial compartmentalization problem is related to the branches of dendrites and the axon terminal. For intracellular stimulation the dendritic tree in FCM was simulated as cylinder with a length equal to the total length of all MCM branches and the diameter was chosen to retain the dendrite surface of the MCM. For extracellular stimulation, the length of the dendrite was shortened as seen in Fig 8 in order to place the center point at a plausible position. The intracellular resistance R needed to solve Eq 4 remained the same as in the intracellular stimulation model. The dendritic membrane voltage of the MCM in Fig 8 corresponds to the branch closest to the electrode. The smaller R of the MCM in comparison to the FCM in Eq 4 explains why the dendrite voltage in the MCM adapted faster than in the FCM (lower part of Fig 8).

For each compartment, the external voltage is determined by the distance of the representative compartment center from the electrode. This means that while a single compartment with a large diameter or length might experience large extracellular voltage differences between different points on its surface, the model only permits one value per compartment. Thus, a finely compartmentalized model simulates the effects of extracellular stimulation more faithfully. In the MCM used here this was not an issue because the cell was compartmentalized and oriented in a way likely to minimize strong extracellular potential differences over the surface of any one compartment. The exclusion of active channels leads to simpler models that make an analysis of effects purely related to cell geometry easier.

The SCM cannot be stimulated with an extracellular electrode and was therefore not included. The experiments were performed with the passive versions of TCM, FCM and MCM. The point source was placed 30 μm above the soma center and a stimulus of 1 μA applied for 1 ms. The membrane voltages were again recorded for soma and axon as well as dendrite and terminal if present.

All cells show the predicted polarization. Due to the missing dendrites, the soma in the TCM is polarized in the opposite way as in the FCM and MCM. The voltage traces of the FCM are roughly comparable to those of the MCM.

#### Active membrane

**Fig 9:**
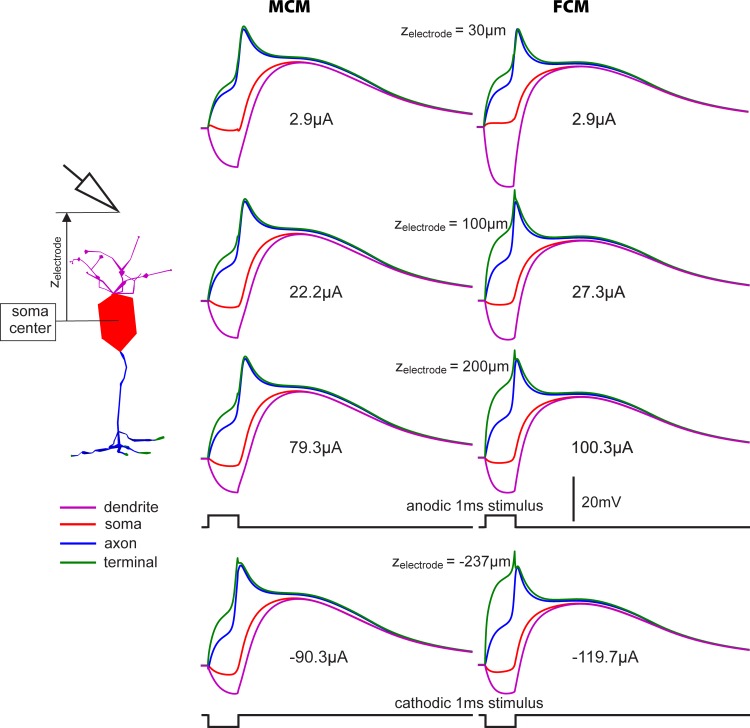
Electrode threshold currents of MCM and FCM generating a spike for four electrode positions along the cell axis. Thresholds (2.9 μA, 22.2 μA, etc.) increased more than linear with z_electrode_, the electrode distance from soma center. Starting at stimulus onset, the membrane voltages were always highest at the terminal, indicating that spikes in the axon were elicited by intracellular current flow from the terminal. Stimulation with anodic 1 ms pulses for the active electrode above the cell and cathodic pulses for electrode positioned below the cell. An electrode distance 30μm simulated a position typical for a subretinal implant, 100 and 200 μm were for epiretinal implants, and the -237 μm case (here the electrode was separated 200 μm from the closest terminal ending) represented suprachoroidal electrode placing [50, 51].

In Fig 8, the electrode distance to a BC of a subretinal implant for blind people is imitated. As the simulated BC type is able to respond with graded potentials as well as with spikes, it is of interest whether spikes can be elicited both for this case as well as for remote electrode positions that are typical for other retinal implant types. Fig 9 shows the membrane voltages Vm of dendrite, soma, axon and terminal as functions of time for stimulus intensities just above threshold for spiking. Starting under resting conditions, the first parts of the four Vm curves are nearly identical, with a response similar to the passive model as long as nearly all ion channel are closed during the subthreshold phase (compare Fig 8 and both graphs at the top of Fig 9 during 1ms of pulse application). During the whole excitation process, the membrane voltage in the terminal is higher than anywhere else in the cell implying that for all shown cases spikes are generated by antidromic current flow from the terminal. This also holds for an electrode position below the cell, where cathodic pulses are needed for spike generation (Fig 9, bottom). For any case shown in Fig 9, the change of polarity causes depolarization of dendrites which is closer to the natural situation when a BC is stimulated via photoreceptors. However, contrary to a synaptic excitation that is strong enough for spiking, extracellular stimulation (which depolarizes dendrites with a comparable amount) hyperpolarizes the axon and terminal strongly enough (simulated but not shown) that no spike can be generated in the axon.

A comparison of the thresholds of MCM and FCM demonstrated that an inaccuracy in the order of around 25% has to be expected for that kind of FCM simulation (Fig 9). The shared 2.9 μA threshold values for the 30 μm distance were due to coincidence and are not an indicator for accuracy as in this case the curves for the dendrite differ strongly and in those for the soma even qualitative differences are apparent during the first millisecond (Fig 9, top).

## Discussion

The FCM was able to produce many of the same results as the MCM for less computational cost and without the need for a very detailed knowledge of the individual cell’s morphology and channel distribution. There were some stimulation tasks for which condensing a system of neurites such as the dendritic tree into a single unit was less suitable. Synapses are located in many different places on the dendritic structure and some are much closer to the soma than others. The same is true for the axon, which is strongly branched in some BCs and connects to different ganglion cells at various path lengths from the soma.

Following the natural signal path through the cell starting with a current influx at a dendritic synapse and ending at one of many axon terminals, the total internal resistance is given by R_Dendrite_+R_Soma_+R_Axon_+R_Terminal_. The axonal and dendritic resistances depend on the position of the respective synapses on the neurites. This is complicated somewhat by the branched structure of the neurites, which influences the resistances as well as transmembrane currents. The MCM was well suited for this situation, the SCM and TCM were not. It was possible to modify the FCM to account for this case by introducing variable resistances for the axon and dendrites (simulated but not shown).

In an exhaustive investigation on rat rod BCs, the following mean parameters for the passive model were found: 1.1 μF/cm^2^ for specific membrane capacitance, 130 Ohm.cm for cytoplasmic resistivity and 0.042 mS/cm^2^ for specific membrane conductance [37]. These values are close to our data (1.0 μF/cm^2^, 100 Ohm.cm, 0.033 mS/cm^2^) based on macaque BCs [9] which were lower than the mean but still within the reported variation for rat neurons [9]. Retinal BCs are among the smallest interneurons of the nervous system. Compared to standard neurons, there is therefore nearly no delay in signaling. Additionally, the small BC surface combined with the low membrane conductance causes very little attenuation in the signal from synaptic input to the terminal. The second fact was shown for passive MCM [37]and demonstrates that there was no need to include active membrane mechanisms for amplification in BCs even for transmitting single photon signals [52].

At a first glance, Fig 5 seems to give the impression that the active channels could not contribute to signal amplification at all because the passive response, which increases linearly with stimulus intensity, was never below that of the active cases. This fact is stressed by the tangent in the middle trace of Fig 5 which has no intersection even when the spike caused a sudden increase. However, the maximum membrane values presented in Fig 5 were for long pulses where the system has settled into something approximating a steady state. For shorter signals the generation of spikes amplified the signal as shown in Fig 4.

In experiments stimulation and recording is often limited to the soma because of its relatively large diameter. This work shows that for passive membranes, the SCM and TCM perform similar to the MCM in this common scenario. Computer simulations can accurately predict currents and activations of voltage-gated channels in locations within the cell distal to the soma.

For computer simulation of BCs even simple models such as single compartment models [4, 21, 22, 31, 53] and two compartment models [23, 26, 33, 51] have been used successfully. In addition to SCM and TCM, multi compartment models in which the spatial structure of the cell is taken into account (2D and 3D approach) have been used for BCs with passive [24, 25, 37–39] and active membranes [27, 40].

A main limitation of SCMs is the neglect of the spatial extend of the non-homogeneous structure which consists of the functionally different segments dendrite, soma, axon and terminal. This limitation becomes obvious for extracellular stimulation which works because the electric field varies along the cell [29]. This variability can be modeled if at least 2 compartments are involved. This is reflected in the circuit diagrams in Fig 2. In the SCM, the extracellular space was on ground potential, in other cases the external voltage was chosen to be zero (ground) for intracellular stimulation or according to the external potential during extracellular stimulation. The BC in this study was recently used to model spike initiation via extracellular microelectrode stimulation [40]. It was shown that there is only a small region for electrode placement where extracellular cathodic stimulation causes direct spike initiation in the axon but for all other positions, a sodium spike can only be generated by antidromic current flow originating from strongly depolarized terminals. Such results, especially quantitatively precise ones require considerably more compartments than the 4 of the presented FCM. Nevertheless, a basic rule for anodic microelectrode stimulation could be confirmed even for the TCM, namely the proximal part of the BC becomes hyperpolarized the other depolarized (Fig 8).

The key element for sodium spikes is a high sodium channel density in the axon [9]. Differing results have to be expected depending on whether these currents are generated in the axon (e.g. FCM) or the soma as in the TCM. The main reason for such differences between models is the intracellular resistance which is especially high for the axon due to its small diameter. The most common methods to evaluate a model is to solve a system of differential equations using a general software such as MATLAB, PYTHON, MATHEMATICA or a specific neuroscience package (NEURON).

The data in this study suggests some heuristics and jump-off points for accuracy tests in future research. For intracellular current injection and passive membranes, even SCMs were adequate. In active membranes, the situation is a bit more complex: nearly all results were qualitatively correct for FCMs but the accuracy depended on the individual task (Figs 4, 5, 7 and 9). Branching dendrites and axon terminals are likely sources of inaccuracy. Better accuracy is expected when each branch distal to a branchpoint is represented with a separate compartment in order to define dendritic and terminal resistances correctly. This problem was demonstrated for the temporal response of the dendrite (MCM vs. FCM) during dendritic stimulation (Fig 4) and for a stimulating microelectrode placed in the vicinity of the dendrite but placing the electrode in the terminal region generates an analogous situation for the branching axon. The next step for an accuracy test could be the usual refinement of spatial discretization until the cell response is constant. Note that neurites are characterized by the space constant λ which is defined by diameter d, intracellular resistivity ϱ_i_ and specific membrane conductance g_m_ as

$$\lambda =\sqrt{d/(4\varrho_{i}g_{m})}$$

Parameters of the actual model with d = 1μm, ϱ_i_ = 0.1kOhm.cm and g_m_ = 0.033mS/cm^2^ result in λ = 86μm. For an upper error limit of 1% a spatial discretization length of λ/4 is recommended [29].

The TCM results often seemed similar to those of the FCM and in one case they were closer to those of the MCM (Fig 3). Closer examination reveals some divergence, e.g. the important sodium currents were substantially lower in the TCM than in the FCM (Figs 4 and 7).

In extracellular stimulation the TCM was very inaccurate even compared to the FCM. To model extracellular stimulation accurately, MCMs are usually necessary; in regions with a strong electric field gradient short compartments are required. The presented model and its previous version are the first attempts to model all key currents involved in a spiking BC. There are however some limitations; while many model parameters are based on the excellent experiments of Puthussery and coworkers [9] some data from other research groups needed to be added to complete the model. For this reason and because some data of the target cell such as resting potential and reversal potentials of the ion currents were not available, the fit was not optimal.

## Appendix model data

The transmembrane voltage *Vn* of the n-th compartment was simulated with

$$C_{n}\frac{{dV}_{n}}{dt}={-I}_{ion,n}-\frac{V_{n}-V_{n-1}}{\frac{R_{n}}{2}+\frac{R_{n-1}}{2}}-\frac{V_{n}-V_{n+1}}{\frac{R_{n}}{2}+\frac{R_{n+1}}{2}}+I_{stim,n}$$

where Cn is the membrane capacitance, *Iion*,*n* is the ionic transmembrane current and Rn is the axial resistance. The second and third terms of the right part of the equation model axial currents between adjacent compartments, the last term is the stimulation current. The units for *V*, *I*, *t* are mV, μA, ms, respectively. See [29] for details.

The ion current term is

$$I_{ion}={(i}_{Na}+i_{Ca,T}+i_{Ca,L}+i_{K,fast}+i_{K,slow}+i_{HCN}+i_{Leakage})A_{n},$$

where the terms within brackets are current densities and *An* is the surface area of the n-th compartment.

In the following equations *m*, *n*, *c and y* are activation variables and *h and s* are inactivation variables. Gating variables of all channels change over time with

$$\frac{dX}{dt}=\frac{X_{\infty }-X}{\tau_{X}}$$

e.g. the equation for *m* is

$$\frac{dm}{dt}=\frac{m_{\infty }-m}{\tau_{m}}$$

and

$$X_{\infty }(V)=\frac{\alpha_{X}(V)}{\alpha_{X}(V)-\beta_{X}(V)}$$

$$\tau_{X}(V)=\frac{1}{\alpha_{X}(V)-\beta_{X}(V)}$$

where α and β are rate coefficients for opening and closing of the ion channel.

The **Sodium Nav1.1** current is given by [45, 46]

$$i_{Na}=g_{Na}m^{3}hs(V-E_{Na})$$

The Nav1.1 density has a maximum gNa = 1000 mS/cm^2^ on the axon at a distance of 22.7 μm from the soma [9], but the sodium band with a surface of 40.8 μm^2^ was modeled with the average values of gNa = 301.4 mS/cm^2^, ENa = 50 mV and *τ*_*m*_ = 0.15 ms. A correction factor was introduced for *h*

$$\tau_{corr,Na}=0.25$$

$$\tau_{h}(V)=\tau_{corr,Na}*20.1*\exp\left(-0.5{\left((V+61.4)/32.7\right)}^{2}\right)$$

$$\tau_{s}(V)=1000*106.7*\exp\left(-0.5{\left((V+52.7)/18.3\right)}^{2}\right)$$

$$m_{\infty }(V)=\frac{1}{1+exp(-(V+27.2)/4.9)}$$

$$h_{\infty }(V)=\frac{1}{1+exp((V+60)/7.7)}$$

$$s_{\infty }(V)=\frac{1}{1+exp((V+60)/5.4)}$$

**Calcium T-type Cav3.1** channels are homogeneously distributed (gCa = 1 mS/cm^2^) in the soma, dendrite and axon hillock compartments. Cav3.1 current kinetics were based on [44]

$$i_{Ca}=g_{Ca}m^{2}h(V-E_{Ca})$$

where ECa = 120 mV and

$$m_{\infty }(V)=\frac{1}{1+exp(-(V+57)/6.2)}$$

$$h_{\infty }(V)=\frac{1}{1+exp((V+81)/4)}$$

$$\tau_{m}(V)=0.612+\frac{1}{\exp\left(-(V+132)/16.7\right)+\exp\left((V+16.8)/18.2\right)}$$

$$\tau_{h}(V)=\left\{ \begin{array}{l} 28+\exp\left(-(V+22)/10.5\right)\;if\;V>-81\;mV\\ \exp\left((V+467)/66.6\right)\;if\;V\leq -81\;mV \end{array}\right.$$

**HCN** current is based on [9, 48], leading to gHCN = 3.52 mS/cm^2^ and

$$i_{HCN}=g_{HCN}y(V-E_{Na})+{\bar{g}}_{HCN}y(V-E_{K})$$

$$\alpha_{y}(V)=\exp\left(-(V+23)/20\right)$$

$$\beta_{y}(V)=\exp\left((V+130)/10\right)$$

The **Calcium L-type** formalism of Cav1.4 current is based on [39] and the kinetics of HCN channels are from [47], leading to gCaL = 11.9 mS/cm^2^

$$i_{Ca}=g_{Ca}c^{3}(V-E_{Ca})$$

ECa = 22.6 mV and

$$\alpha_{c}(V)=\frac{0.4(V+80)}{1-exp(-0.1(V+88))}$$

$$\beta_{c}(V)=10\;\exp\left(-(V+76)/12.6\right)$$

**Potassium** currents are based on the original Hodgkin-Huxley potassium channel model. The slow potassium channels are located at soma, dendrite, and axon hillock with gKslow = 2.4 mS/cm^2^ and the kinetics are governed by

$$i_{K}=g_{K}n^{4}(V-E_{K})$$

EK = -77 mV

$$\tau_{corr,Kslow}=8$$

$$\alpha_{n,slow}(V)=\frac{0.01(V+55)}{\tau_{corr,Kslow}exp(-0.1(V+55))}$$

$$\beta_{n,slow}(V)=0.125\frac{\exp\left(-(V+65)/80\right)}{\tau_{corr,Kslow}}$$

The fast type potassium channels are located in the sodium band (the part of the axon containing a high density of sodium channels) with gKfast = 2 mS/cm^2^

$$V_{offset,fast}=5\;mV$$

$$\tau_{corr,Kfast}=5$$

$$\alpha_{n,fast}(V)=\frac{0.01(V-V_{offset,fast}+55)}{\tau_{corr,Kfast}exp(-0.1(V-V_{offset,fast}+55))}$$

$$\beta_{n,fast}(V)=0.125\frac{\exp\left(-(V-V_{offset,fast}+65)/80\right)}{\tau_{corr,Kfast}}$$

All calculations were done at 31°C, consistent with [9]. To adjust for this, the rate coefficients α and β, which were measured at temperature T1, were multiplied by Q10^((T2-T1)/10) with the temperature coefficient Q10 and T2 = 31°C. Initial temperatures for sodium, potassium, T-type calcium, L-type calcium, and HCN were 20, 6.3, 24, 10, 37°C, respectively [38, 43–45, 48]. The following values for Q10 were used [30, 44, 48] *Q*_10,*m*,*Na*_ = 2.2, *Q*_10,*h*,*Na*_ = 2.9, *Q*_10,*s*,*Na*_ = 2.9, *Q*_10,*m*,*CaT*_ = 5, *Q*_10,*h*,*CaT*_ = 3, *Q*_10,*y*,*HCN*_ = 1, *Q*_10,*c*,*CaL*_ = 1, *Q*_10,*n*,*kfast*_ = 2, *Q*_10,*n*,*kslow*_ = 2.

## Abbreviations

BC: bipolar cell
FCM: four compartment model
MCM: multicompartment model
SCM: single compartment model
TCM: two compartment model
Ve: extracellular potential
